# The Natural Antimicrobial Carvacrol Inhibits Quorum Sensing in *Chromobacterium violaceum* and Reduces Bacterial Biofilm Formation at Sub-Lethal Concentrations

**DOI:** 10.1371/journal.pone.0093414

**Published:** 2014-04-01

**Authors:** Sara A. Burt, Victoria T. A. Ojo-Fakunle, Jenifer Woertman, Edwin J. A. Veldhuizen

**Affiliations:** 1 Institute for Risk Assessment Sciences, Veterinary Public Health Division, Faculty of Veterinary Medicine, Utrecht University, Utrecht, The Netherlands; 2 Department of Infectious Diseases and Immunology, Faculty of Veterinary Medicine, Utrecht University, Utrecht, The Netherlands; University of Manchester, United Kingdom

## Abstract

The formation of biofilm by bacteria confers resistance to biocides and presents problems in medical and veterinary clinical settings. Here we report the effect of carvacrol, one of the major antimicrobial components of oregano oil, on the formation of biofilms and its activity on existing biofilms. Assays were carried out in polystyrene microplates to observe (a) the effect of 0–0.8 mM carvacrol on the formation of biofilms by selected bacterial pathogens over 24 h and (b) the effect of 0–8 mM carvacrol on the stability of pre-formed biofilms. Carvacrol was able to inhibit the formation of biofilms of *Chromobacterium violaceum* ATCC 12472, *Salmonella enterica* subsp. Typhimurium DT104, and *Staphylococcus aureus* 0074, while it showed no effect on formation of *Pseudomonas aeruginosa* (field isolate) biofilms. This inhibitory effect of carvacrol was observed at sub-lethal concentrations (<0.5 mM) where no effect was seen on total bacterial numbers, indicating that carvacrol's bactericidal effect was not causing the observed inhibition of biofilm formation. In contrast, carvacrol had (up to 8 mM) very little or no activity against existing biofilms of the bacteria described, showing that formation of the biofilm also confers protection against this compound. Since quorum sensing is an essential part of biofilm formation, the effect of carvacrol on quorum sensing of *C. violaceum* was also studied. Sub-MIC concentrations of carvacrol reduced expression of *cviI* (a gene coding for the N-acyl-L-homoserine lactone synthase), production of violacein (pigmentation) and chitinase activity (both regulated by quorum sensing) at concentrations coinciding with carvacrol's inhibiting effect on biofilm formation. These results indicate that carvacrol's activity in inhibition of biofilm formation may be related to the disruption of quorum sensing.

## Introduction

The formation of biofilms by bacteria confers resistance to biocides and represents a serious problem in medical and veterinary clinical settings [1]. Bacterial biofilm activity is regulated by quorum sensing (QS), a system used by both Gram positive and Gram negative bacteria based on the secretion and/or detection of external signal molecules; QS also influences motility and the expression of flagella [2]–[3]. Finding new ways to target QS in bacteria is an acknowledged strategy for finding and developing new antibiotics [4].

Some essential oils have been shown to inhibit QS in bacteria and this has been proposed as a mechanism of their antibacterial activity [5]. Previous studies have shown that sub-lethal concentrations of carvacrol, a component of oregano essential oil, reduce motility and invasiveness in bacteria [6]–[7]. This antimicrobial compound can reduce biofilm formation in staphylococci and *Salmonella* strains [8]–[9]. The mechanism by which carvacrol inhibits biofilm accretion has not yet been fully established.

From our previous studies showing that carvacrol reduces bacterial motility and virulence at sub-lethal concentrations [6]–[7], we hypothesised that carvacrol may interfere with the QS signalling mechanism between bacterial cells, thereby also reducing the capacity for biofilm formation. This idea is supported by a report that an *Escherichia coli* mutant lacking qseC, a membrane protein involved in QS, was more sensitive to carvacrol than the wild type [10].

The aim of this study was to evaluate whether carvacrol could influence biofilm formation in selected bacterial pathogens or could break down an established biofilm. The role of carvacrol in interference in the bacterial QS mechanism was also investigated. The study shows that carvacrol can inhibit the formation of bacterial biofilms and disrupts quorum sensing at sub-lethal concentrations but has no apparent effect on established biofilms.

## Materials and Methods

### Bacterial strains and growth conditions

The bacterial strains used were *Chromobacterium violaceum* ATCC 12472, *Salmonella enterica* subsp. Typhimurium DT104, *Staphylococcus aureus* BMA/FR/032/0074 and *Pseudomonas aeruginosa* (field isolate). Before experiments, bacteria were inoculated into broth and cultured 16 h as follows: *S.* Typhimurium and *P. aeruginosa* in tryptic soy broth (TSB) at 37°C, *S. aureus* in TSB + 0.25% glucose (TSBG) at 37°C; *C. violaceum* in Luria Bertani broth (LB) at 26°C.

Colony counting was carried out when necessary by serial decimal dilution in phosphate-buffered saline (PBS) and plating out on tryptic soy agar (TSA) and incubation at the appropriate temperature for 20 h (48 h for *C. violaceum*).

### Biofilm inhibition assay

The assay method of Molhoek et al. [11] was used, with optimization of media and temperature for the different bacterial strains. Carvacrol (98% pure, Sigma-Aldrich) solutions were prepared in TSB (1/20 dilution TSB for *S.* Typhimurium) from a stock solution in ethanol. The outer rows and columns of a flat bottomed medium binding 96-wells plate (Greiner Bio-one 655101) were filled with sterile PBS to prevent evaporation from the central wells. Carvacrol concentrations from 0 mM (ethanol as vehicle, negative control) to 2.0 mM carvacrol were arranged horizontally across the six remaining rows (50 μl per well). Sixteen hour bacterial cultures were adjusted to OD (590 nm) 0.02 and aliquots of 50 μl were added to the test wells, leaving one column for sterile broth only (blank). Plates were incubated 24 h on a shaker at 600 rpm at 37°C (*S. aureus*, *P. aeruginosa*) or 26°C (*C. violaceum*), or without shaking at 25°C (*S.* Typhimurium). Wells were emptied and washed twice with PBS to remove loose bacteria. *S. aureus* biofilms were immediately stained using 0.4% crystal violet (Boom, colour index 42555) for 5 min. For *S.* Typhimurium and *P. aeruginosa* the biofilm was first fixed by adding 100 μl methanol to each well for 15 min and then stained using 0.4% crystal violet (Klinipath, colour index 42555). Excess stain was removed by rinsing three times with PBS. Bound crystal violet was solubilized by adding 100 μl 33% acetic acid per well and after shaking the OD at 590 nm was measured. The mean of the six replicates were calculated after subtraction of the blank measurement and the results were expressed as a percentage biofilm in relation to the untreated control. Each assay was carried out three or four times.

### Pre-formed biofilm destruction assay

A 16 h bacterial culture was adjusted to an OD 590 nm of 0.01 using the appropriate growth medium and aliquots of 100 μl were added to two 96-wells plates. The outer rows and columns of the plates contained PBS to prevent dehydration, and the same incubation conditions were used. The next day, the bacterial suspension was discarded and the wells were rinsed three times with PBS to remove loose bacteria. One plate was used to measure the extent of the biofilm by staining with crystal violet; the other plate was used to evaluate the effect of carvacrol on the biofilm. Carvacrol concentrations of 0 mM (ethanol as vehicle), 2, 4, 6, and 8 mM in distilled water were added to the pre-formed biofilm in the wells. One column received 100 μl sterile distilled water as an extra negative control. Plates were incubated 24 h at 20°C without shaking. The well contents were then discarded, wells rinsed three times with PBS and staining was carried out as before. The mean of the six replicates were calculated after subtraction of the blank measurement and results were expressed as a percentage of the untreated 24 h biofilm. Assays were carried out three times.

### Quantitative analysis of *cviI* gene expression

The effect of carvacrol on the expression of the *cviI* gene, that codes for the N-acyl-L-homoserine lactone (AHL) synthase, was measured using quantitative PCR. A 16 h culture of *C. violaceum* was diluted to 2×10^5^ cfu/ml in LB. Carvacrol was added to a final concentration of 0, 0.2 or 0.3 mM and bacteria were cultured at 26°C. One ml samples were taken after 24, 48 and 72 h. Bacteria were centrifuged and resuspended in 1 ml TRIzol (Invitrogen life technologies, Carlsbad, CA). Total cellular RNA was isolated according to the supplier's recommendation. Purity and quality of the RNA extracts was checked on 1% agarose gels and using UV absorption at 260/280 nm. Approximately 500 ng RNA was used to produce cDNA, using iScript (Bio-rad Laboratories BV, Veenendaal, The Netherlands) according to the manufacturer's recommendations. Quantitative determination of gene expression of *cviI* was determined using quantitative polymerase chain reaction according to the following cycle protocol: 10 min at 95°C (denaturation), 40 cycles: 10 s at 95°C, 60 s at 60°C. The 16S Ribosomal RNA gene was used as reference gene. Results are expressed as fold increase compared to the control (0 mM Carvacrol) sample at t = 24 h of three independent experiments. Primers for *cviI* and the housekeeping gene 16S ribosomal RNA are depicted in Table 1.

**Table 1:** The quantitative real-time RT-PCR primers used in this study.

cviI	Forward	5′CTGAAACTAAGCTGCGACAGTTG 3′
	Reverse	5′GAAACCGTCCTCGCATAAGG 3′
16S	Forward	5′ GCGCAACCCTTGTCCTTAGTT 3′
	Reverse	5′TGTCACCGGCAGTCTCCTTAG 3′

### Quorum sensing inhibition assay

This assay measured the amount of violacein, a violet pigment, produced by *C. violaceum* as a result of QS activity [12]. Carvacrol stock solution was added to 10 ml portions of LB in screw-capped glass bottles so as to achieve concentrations in a range from 0–0.8 mM carvacrol. All bottles were inoculated with 10 μl of *C. violaceum* (16 h culture adjusted to 0.01 OD in LB) and incubated 72 h at 26°C. The numbers of cfu/ml in the original inoculate were confirmed and cfu/ml after incubation in the presence of carvacrol were determined by decimal dilution and plating out on agar. From each 72 h sample, a 4 ml portion was centrifuged 3 min at 13000 *g* and the supernatant was discarded. Violacein was extracted from the samples according to the method of Singh et al. [12]. In brief, the pellet was resuspended in 200 μl LB, to which 200 μl 10% SDS was added to lyse the bacteria. After 5 min at room temperature, 900 μl water-saturated *n*-butanol was added, vortexed to dissolve the violacein present, and centrifuged 5 min at 13000 *g*. Supernatants were transferred to a 96-wells plate and the OD was measured at 590 nm on a plate reader. A blank reading (sterile LB) was subtracted from the measurements. The assay was carried out three times.

### Determination of chitinolytic activity

Chitinolytic activity was determined as described [13]. Agar plates were prepared containing 0.4% K_2_HPO_4_ (w/v), 0.2% KH_2_PO_4_ (w/v), 0.15% MgSO_4_•7H_2_O (w/v), 0.1% NaCl (w/v), 0.01% yeast extract (w/v), and 1.0% chitin(w/v) (C7170, Sigma-Aldrich), 1.5% agar (w/v) and 0–0.5 mM carvacrol. Plates were inoculated with a 16 h culture of *C. violaceum* using a sterile toothpick and were incubated for 14 d at 26°C. The diameter of the transparent zones which formed around the *C. violaceum* colonies due to the breakdown of chitin were measured using a ruler. Three independent experiments were performed in triplicate.

### Statistical analysis

Results of assays were compared using SPSS software (version 20). A Kruskal-Wallis analysis of variance was carried out followed post-hoc by the Mann-Whitney U test to compare treatment concentrations with the control. Differences were stated as statistically different if *p* was less than 0.05.

## Results and Discussion

### Carvacrol reduces biofilm formation by *C. violaceum*, *S. Typhimurium* and *S. aureus* but has no effect on *P. aeruginosa* biofilms formation

**Figure 1:**
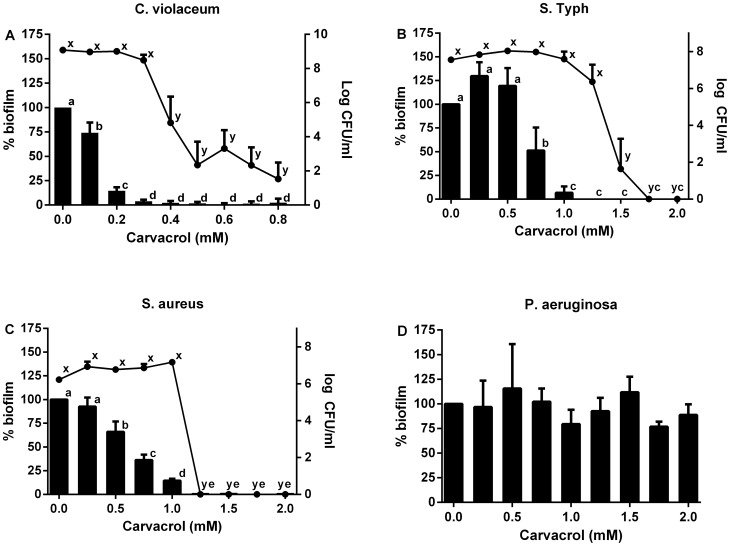
(A–D). Carvacrol reduces biofilm formation by *C. violaceum*, *S. Typhimurium* and *S. aureus* but not *P. aeruginosa*. Percentage biofilm formation measured as OD (590 nm) after crystal violet staining after 24 h incubation in the presence or absence of carvacrol (0–0.8 mM) (bars, left axis) compared to colony counts (log cfu/ml) from the same samples (connected dots, right axis). *C. violaceum* (A), S. Typhimurium (B), *S. aureus* (C), *P. aeruginosa* (D). Data points which are significantly different from each other are denoted by different letters (*p*<0.05). Bars indicate standard error of the mean.

The effect of carvacrol on the ability of three bacterial pathogens and *C. violaceum* to form biofilms was measured in a microplate model. Viable numbers of bacteria after biofilm formation were also determined using colony counting. The results for the biofilm inhibition assays, presented in Figure 1, showed that for *C. violaceum*, *S.* Typhimurium and *S. aureus* a significant reduction in biofilm formation was found between 0.1–0.3 mM for *C. violaceum*, between 0.75–1.25 mM for *S.* Typhimurium, and between 0.50–1 mM for *S. aureus* (all *p*<0.05). At these concentrations of carvacrol no reduction in bacterial counts were observed indicating that bacterial killing was not responsible for the lowered biofilm formation. For example in Fig. 1a the biofilm formation of *C. violaceum* is significantly reduced at 0.1 mM carvacrol and absent from 0.3 mM while a significant reduction in bacterial counts is not observed until the concentration of carvacrol reaches 0.4 mM and above. For *P. aeruginosa* no significant inhibition of biofilm formation was measured in the presence of up to 2.0 mM carvacrol (Fig. 1d), showing that this bacterium is resistant to the biofilm inhibiting activity of carvacrol.

These results corroborate the work of other researchers who found that non-biocidal concentrations up to 0.012% (approx. 0.8 mM) carvacrol significantly reduced biofilm formation in three strains of *S.* Typhimurium (9). Carvacrol at 0.5 MIC (approx. 1 mM) was also effective at reducing the amount of *S. aureus* and *S. epidermidis* biofilms on polystyrene (8). Since biofilm development can be inhibited without reducing bacterial viability, it seems likely that a mechanism other than growth inhibition or bacterial cell death may be involved in carvacrol's antibiofilm activity.

### Carvacrol has no significant effect on pre-formed biofilms

**Figure 2:**
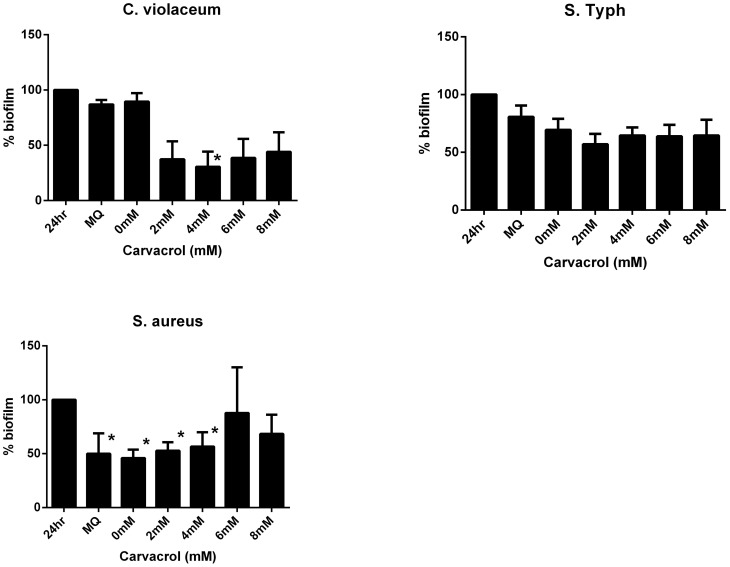
(A–C). Carvacrol has no significant effect on pre-formed biofilms. Percentage remaining of pre-formed biofilms after 24 h incubation in presence or absence of carvacrol (0–8 mM). Expressed as percentage original biofilm measured as OD at 590 nm. *C. violaceum* (A), *S.* Typhimurium (B), *S. aureus* (C). Data points marked with asterisks are just significantly lower than the 24 h control (*p* = 0.05). Bars indicate standard error of the mean.

The effect of a range of carvacrol concentrations on the thickness of a pre-formed (24 h) biofilm was determined in a microplate model. The results showed that for *C. violaceum*, *S.* Typhimurium, and *S. aureus* a small but not significant reduction in pre-existing biofilm was achieved by treatment with up to 8 mM carvacrol over 24 h (Fig. 2). Although some points in Fig. 2 are associated with a *p* value of exactly 0.05, which is just outside the range of significant difference, there was no correlation between biofilm reduction and increasing carvacrol concentration. The observed reduction therefore seems to be a random observation due to extra handling (washing steps) of the biofilm compared to the 24 h biofilm. Indeed, except for *C violaceum* biofilms, treatment of the biofilm with sterile distilled water also resulted in removal of biofilm.

*P. aeruginosa* was not tested in this assay because under our experimental conditions these films were unstable for longer incubation times and spontaneous removal from the plastic was observed. However, since carvacrol did not reduce biofilm formation for this strain it was assumed that it would also be ineffective against pre-formed biofilms.

In contrast, an earlier study found 0.025% (approx. 1.7 mM) carvacrol to be significantly useful in reducing pre-formed biofilms of *Listeria monocytogenes* [14]. Bacterial species susceptibility as well as methodological differences may account for this contrast since biofilm mass was assessed after counting culturable bacteria released by swabbing [14].

Markedly higher concentrations of carvacrol than have been used in this study have been shown to be helpful in reducing biofilms. From 0.3% w/w (approx. 20 mM) carvacrol dissolved in a surfactant solution was useful in reducing *E. coli* biofilms on steel coupons [15]. Staphylococcal biofilms were reduced after treatment with 1% v/v (approx. 66 mM) carvacrol [16]. Daily pulses of 20 mM carvacrol (representing 1 mmol/h) were successful in inhibiting *S. aureus* biofilm accumulation in a fermentor system but were not very effective against *S.* Typhimurium biofilm [17].

### Carvacrol inhibits gene expression of *cviI* at concentrations too low to influence bacterial growth or survival

**Figure 3:**
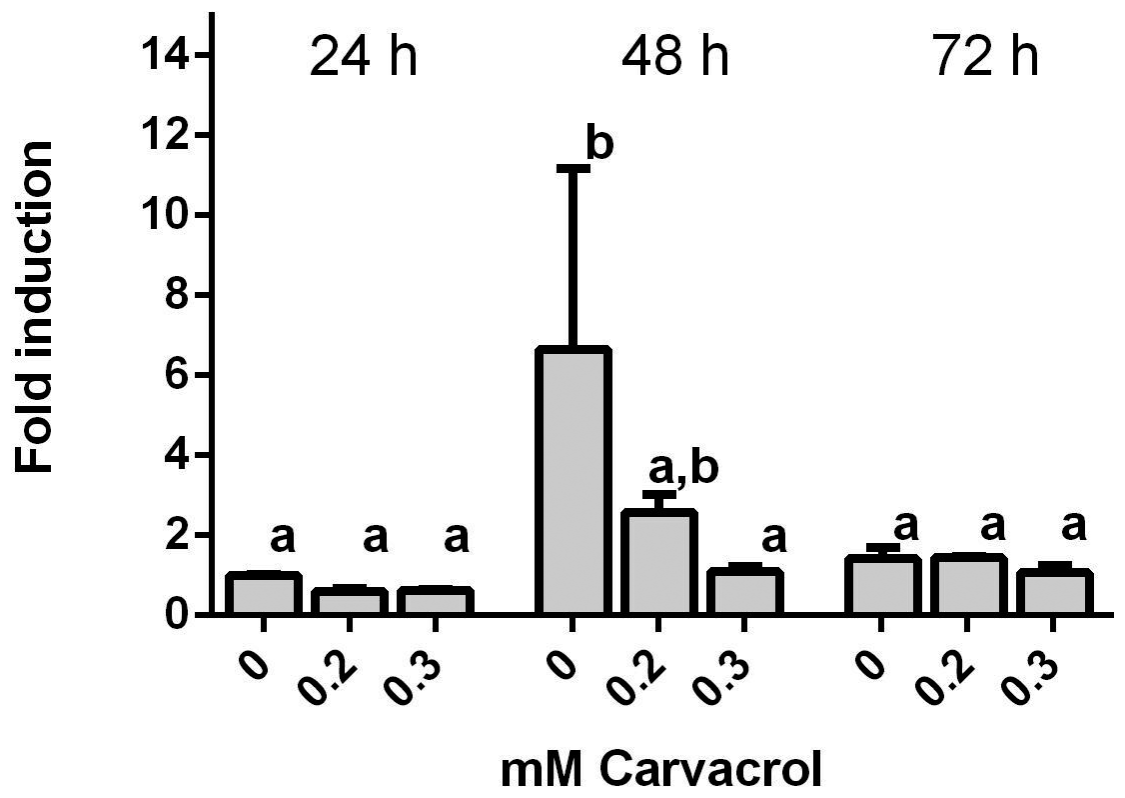
Carvacrol inhibits gene expression of *cviI* at concentrations too low to influence bacterial growth or survival. Expression of *cviI* by *C. violaceum* after incubation with carvacrol for 24, 48 and 72 h. Mean fold increases compared to t = 24 h, 0 mM carvacrol for three experiments are shown. Data points which are significantly different from each other are denoted by different letters (*p*<0.05). Bars indicate standard error of the mean.

Since biofilm formation is known to be regulated by quorum sensing, further studies were performed to establish the correlation between carvacrol treatment and QS in *C. violaceum*. The effect of carvacrol on the *cviI* gene coding for the N-acyl-L-homoserine lactone (AHL) synthase was measured using quantitative PCR. Growth of *C. violaceum* at 26°C in the absence of carvacrol resulted in a significant (p<0.05) 6-fold increase of *cviI* gene expression after 48 h compared to 24 h and the level dropped back to original levels after 72 h (Fig. 3). Similarly, in the presence of 0.2 mM carvacrol, a 2-fold increase was observed at 48 h, however, 0.3 mM carvacrol inhibited the *cviI* gene expression at 48 h growth. These results indicate that carvacrol inhibited the production of AHL at the level of gene expression of its synthase gene. This appears to be the first report confirming that carvacrol affects genes coding for QS.

### Carvacrol inhibits QS-related violacein production by *C. violaceum*

**Figure 4:**
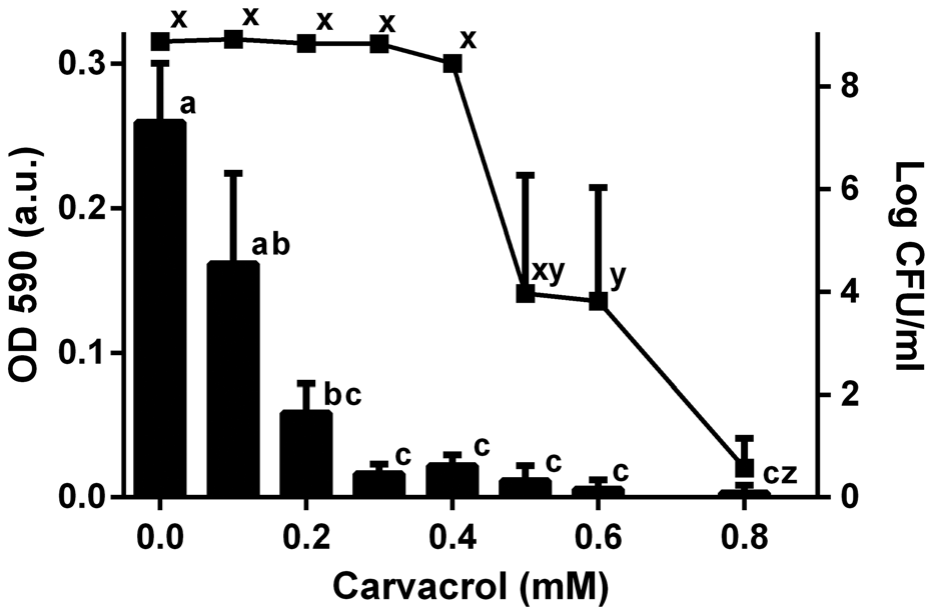
Carvacrol inhibits production of violacein by *C. violaceum*. The amount of violacein pigment present in a butanol extraction after 24(0–0.8 mM) measured as OD 590 nm (bars, left axis), compared to the colony count of bacteria present in the suspension (log cfu/ml) (connected dots, right axis). Data points which are significantly different from each other are denoted by different letters (*p*<0.05). Bars indicate standard error of the mean.

The effect of a range of carvacrol concentrations on the production of a QS-related pigment by *C. violaceum* was measured and compared with the concentration of carvacrol required to reduce the number of viable bacteria present. The results are presented in Fig. 4. From >0.1 mM carvacrol a significant reduction in the production of violacein pigment was measured (*p*<0.05), whilst the numbers of cfu/ml present remained constant up to at least 0.4 mM carvacrol. The reduction in violacein at 0.1–0.4 mM carvacrol indicates that carvacrol can inhibit bacterial QS at these concentrations. This concentration range agrees closely with the carvacrol concentrations at which *C. violaceum* showed reduced biofilm formation (Fig. 1a) and reduced *cviI* gene expression (Fig. 3), indicating that the mechanism by which carvacrol inhibits biofilm formation may be linked to the disruption of QS in bacteria. This phenomenon may be useful in reducing virulence of antimicrobial resistant bacterial strains, e.g. in combination with antibiotics [1], [4], [18].

### Carvacrol inhibits chitinolytic activity of *C. violaceum*

**Figure 5:**
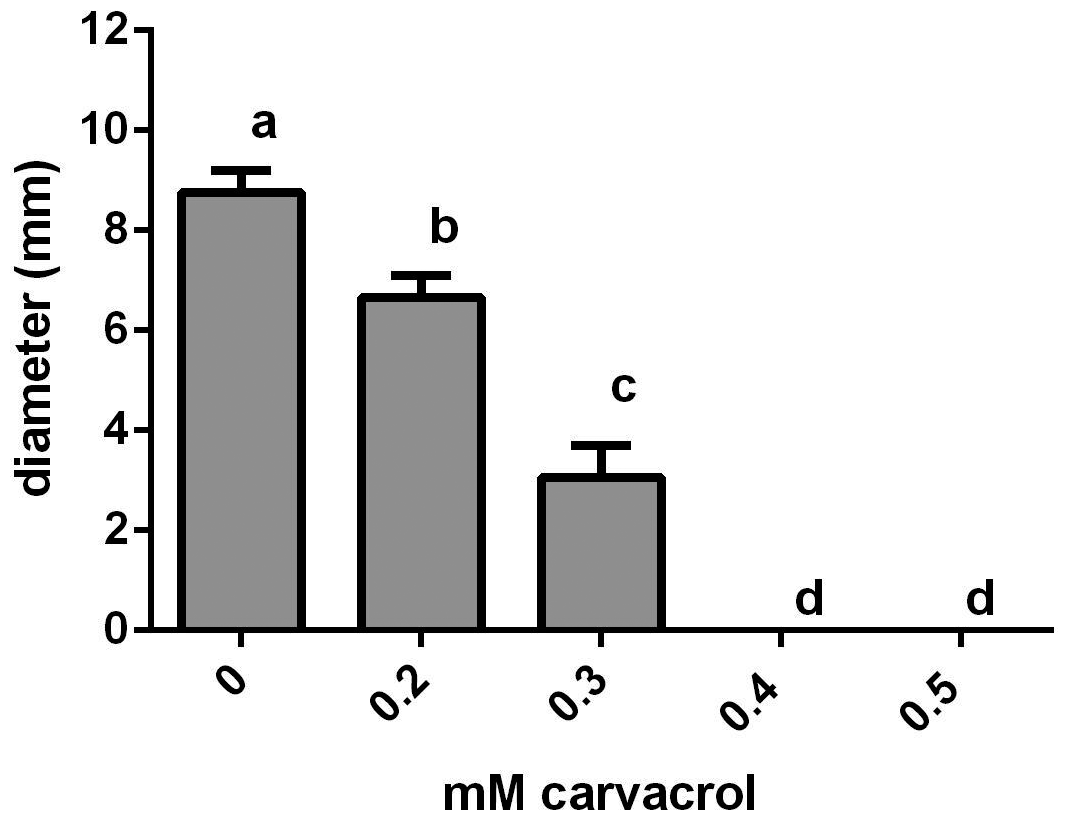
Carvacrol inhibits chitinolytic activity of *C. violaceum*. Diameter of clear chitinolytic zone around *C. violaceum* centrally inoculated onto low-nutrient agar plates containing chitin after 14 d incubation (mean of three experiments). Data points which are significantly different from each other are denoted by different letters (*p*<0.05). Bars indicate standard error of the mean.

A third trait of *C. violaceum* regulated by QS is its ability to increase chitinase production in the presence of chitin in a low nutrient environment [19]. Chitinase activity was determined by inoculating bacteria onto agar-chitin plates supplemented with 0–0.5 mM carvacrol. After incubation at 26°C for 14 d the clearing zone around the bacterial colonies was measured with a ruler. The average diameter of the clearance zones was significantly reduced at 0.2 and 0.3 mM carvacrol and absent at higher carvacrol concentrations (Fig. 5).

The described results clearly show that for *C. violaceum* carvacrol reduces the gene expression of the AHL synthase *cviI* and three QS related traits: biofilm formation, violacein production and chitinase production. Genes controlling these processes are all under the control of *cviR*, the cytoplasmic DNA response element that binds the C10-HSL molecule produced by AHL-synthase. However, the *cviI* gene itself is also under the control of *cviR* [20] therefore the observed decrease in *cviI* gene expression could be a secondary effect of carvacrol' s quorum sensing inhibiting activity.

Inhibition of QS by phytochemicals has been described before [21]–[22] indicating that a general mechanism may apply, rather than binding to a clearly specified molecular target. Similarly, the effect of carvacrol on biofilm formation of three bacterial strains suggests a more general mechanism than binding of carvacrol to *cviR* or C10-HSL itself. The *cviI*/*cviR* system of *Chromobacter* spp. is homologous to *luxI/luxR* used by many Gram negative bacteria. *Salmonella* can detect AHLs but not synthesise it due to the lack of a *luxI* homologue. In contrast, *P. aeruginosa* possesses both components of the *LuxI/R* system [23] but biofilm formation was not affected by carvacrol in our experimental set-up. *S. aureus* uses a completely different QS system (the *agr* system) in which the effector molecule is actually a small RNA (RNAIII). It is therefore hard to explain the observed effects of carvacrol on the tested bacteria using different QS systems.

One comparable feature of carvacrol and many other phytochemicals is that their initial target is likely the bacterial membrane. At MIC values complete leakage and loss of membrane potential is observed [24]. It is possible that at lower concentrations smaller destabilisation effects of these membranes occur which could reduce the QS capability of bacteria. At concentrations that do not cause sufficient leakage to actually kill bacteria, loss of ions and ATP, reduced membrane potential or other physiological changes could for example affect gene expression of AHL synthases and AHL receptors, or AHL-synthase activity. Further in depth studies on carvacrol's activity to inhibit QS are required to shed light on its exact mechanism of action.

## Conclusion

This study shows that carvacrol inhibits the formation of biofilms by selected bacterial pathogens at concentrations which do not impair bacterial growth or survival. The anti-biofilm activity of carvacrol appears to be related to interference with bacterial QS since other QS-induced phenomena such as chitinase production and violacein production in *C. violaceum* are also affected.
